# Protocol to chemically deplete phagocytic hemocytes in *Anopheles gambiae* using clodronate liposomes

**DOI:** 10.1016/j.xpro.2025.103819

**Published:** 2025-05-15

**Authors:** Hyeogsun Kwon, Ryan C. Smith

**Affiliations:** 1 Department of Plant Pathology, Entomology, and Microbiology, Iowa State University, Ames, IA 50011, USA

**Keywords:** Cell separation/fractionation, Genetics, Immunology, Model Organisms, Molecular Biology, Special Issue, Protocols in Entomology

## Abstract

Understanding the roles of phagocytic hemocytes in mosquito innate immunity has been significantly limited due to the lack of genetic tools. Here, we present a protocol for depleting phagocytic hemocytes in *Anopheles gambiae* mosquitoes using clodronate liposomes. We describe steps for mosquito injection, as well as validation by microscopy, quantitative real-time PCR (real-time qPCR), and flow cytometry analysis. This protocol allows for the delineation of phagocytic hemocyte function in mosquito immunity, which can be more broadly applied to other arthropod systems.

For complete details on the use and execution of this protocol, please refer to Kwon et al.^1^

## Highlights

• Techniques to deplete phagocytic immune cells in mosquitoes
• Microscopy-based approaches to validate phagocytic granulocyte depletion
• Molecular confirmation of phagocytic granulocyte depletion using real-time qPCR
• Use of flow cytometry to examine phagocytic granulocyte depletion

## Abstract (editor highlights)

Publisher’s note: Undertaking any experimental protocol requires adherence to local institutional guidelines for laboratory safety and ethics.

## Graphical abstract

**Figure:**
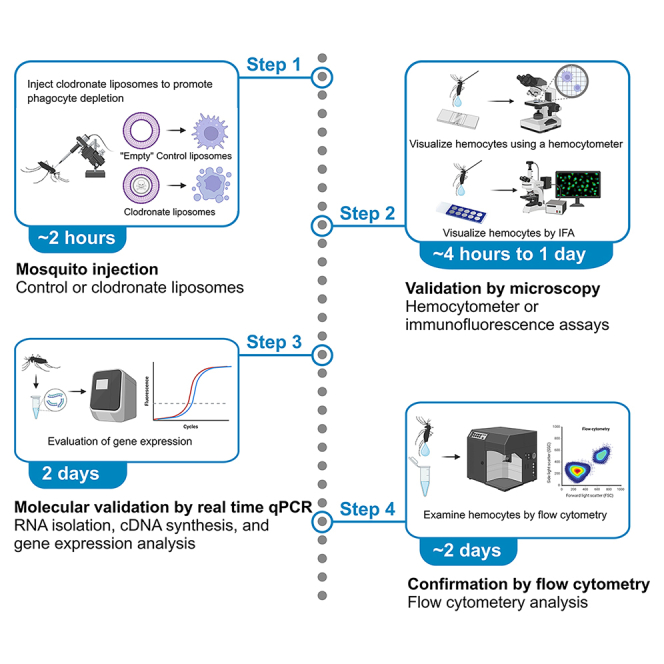

## Before you begin

Our protocol describes the application and use of clodronate liposomes to study mosquito immune cell function in *Anopheles gambiae*,^1^ although similar approaches have been recently applied in other arthropod systems.^2^^,^^3^^,^^4^^,^^5^ Herein, we outline procedures for liposome injection and subsequent methods to validate phagocyte depletion using microscopy, gene expression, and flow cytometry techniques.

### Institutional permissions

Research involving mosquitoes and their containment in the laboratory should be performed according to Arthropod Containment Guidelines^6^ with Institutional Biosafety oversight. Additional complementary studies with mosquito-borne pathogens should be performed with proper biosafety containment and may require additional approvals by Institutional Biosafety or Institutional Animal Care and Use Committees.

### Mosquito rearing

**Timing: ∼2 weeks to go from eggs to adult mosquitoes**

*Anopheles gambiae* mosquitoes can be reared under laboratory conditions using a variety of methods and equipment.^7^ This includes differences in larval diet and in providing blood meals that are required for reproduction and the propagation of the colony using vertebrate animals (such as mice) or through artificial membrane feeding with commercially provided blood. In addition, mosquitoes can be maintained at temperatures between 25°C–27°C, ∼70-80% humidity, and between 12–14 h of daylight. For this protocol, the Keele strain of *An. gambiae*^8^ was provided as larvae with ground fish flakes (Tetramin; Tetra), with adults maintained on 10% sucrose and commercial sheep blood via an artificial membrane feeder for egg production. Mosquitoes were reared at 27°C, 80% relative humidity, and a 14/10-h day/night cycle throughout development.

### Preparation of needles for injections and hemolymph perfusion

**Timing: ∼15 min**

1. Prepare needles for hemolymph perfusion (to collect hemocytes) using glass capillary tubes and a micropipette puller (Sutter Instrument P-97 or P-2000).
  a. Settings for pulling needles using a P-97 puller are listed below. This should be considered a starting point since there will be some variance in the settings between instruments and each time that the heating filament is changed.

**Table:** 

Heat	Pull	Velocity (VEL)	Time	Pressure (P)
320	100	100	200	500

***Note:*** It is essential to adjust the parameters of the micropipette puller to create needles with an optimal taper and diameter to maximize flow rate and to ensure survival after injection. Liquid should “bubble” out of the needle only following the applied pressure of the injection and should not leak. Use the recovery of mosquitoes after injection with 1X PBS or other inert buffer as an additional quick readout to assess needle quality. An “ideal” needle can only be determined through learning from trial and error.

2. Pull an adequate number of needles (5–10) prior to an experiment such that they can easily be replaced if one becomes clogged or broken.
3. Immediately before use, break the tip of the needle under a microscope using forceps. Test the flow rate prior to use.

### Preparation for hemolymph perfusion

**Timing: ∼30 min**

Prepare an anticoagulant buffer (pH of ∼5.5–6.0) solution to enhance hemolymph perfusion and hemocyte collection. Batches ranging in size from 10 to 100 ml can be prepared, aliquoted in 1.5 ml tubes, and stored at −20°C until later use. Details for making the anticoagulant buffer are displayed under the materials and equipment heading below.

### Design and ordering of PCR primers

**Timing: ∼2–3 days for delivery**

4. Methods to confirm phagocytic hemocyte depletion by quantitative real-time PCR (real-time qPCR) require gene-specific and internal standard primers. Existing primers targeting *Eater* or *Nimrod B2* can be used by proxy to examine phagocytic granulocyte numbers,^1^ *PPO1* can be used as a hemocyte marker (oenocytoid) for non-target cell types,^9^^,^^10^ with *rpS7* serving as an internal control.^1^^,^^9^^,^^10^ Oligonucleotide primers can be synthesized by a variety of sources, including Thermo Fisher Scientific which was used in our studies.

***Note:*** Additional candidate genes can potentially be developed to serve as markers of *An. gambiae* hemocyte subtypes based on previous proteomic^11^ and single-cell studies.^9^^,^^12^

## Key resources table

**Table:** 

REAGENT or RESOURCE	SOURCE	IDENTIFIER
**Chemicals, peptides, and recombinant proteins**		

Sigmacote	Sigma-Aldrich	SL2-25ML
Fetal bovine serum (BSA)	Thermo Fisher Scientific	A5256701
Citric acid	Fisher Scientific	A104-500
Sodium hydroxide	Fisher Scientific	S318-500
Sodium chloride	Fisher Scientific	163664
Ethylenediaminetetraacetic acid (EDTA)	Sigma-Aldrich	E6758
Phosphate-buffered saline (PBS)	Gibco	2797883
4% paraformaldehyde	Affymetrix	2265C323
Mineral oil	MP Biomedicals	194836
TRIzol	Thermo Fisher Scientific	15596018
Chloroform	Thermo Fisher Scientific	C/4960/17
Isopropyl alcohol	Fisher Scientific	BP2618212
Ethyl alcohol	Sigma-Aldrich	459828-2L
RNase-free water	Thermo Fisher Scientific	10977015

**Experimental models: Organisms/strains**		

*Anopheles gambiae*	4- to 6-day-old adult females	Keele strain

**Oligonucleotides**		

Gene-specific qPCR primers	Eater-qF: 5′- TTCACCCGTCTGCGAGGGATGCAAGC-3′ Eater-qR: 5′- GTCAACGTGCATAGTAGCGTCTCCGTAGC-3′ Nimrod B2-qF: 5′-CAATCTGCTCAAATGGCTGCTTCCACG-3′ Nimrod B2-qR: 5′- GCTGCAAACATTCGGTCCAGTGCATTC-3′ PPO1-qF: 5′- GACTCTACCCGGATCGGAAG-3′ PPO1-qR:5′- ACTACCGTGATCGACTGGAC-3′ rpS7-qF: 5′- ACCACCATCGAACACAAAGTTGACACT-3′ rpS7-qR: 5′- CTCCGATCTTTCACATTCCAGTAGCAC-3′	AGAP012386 AGAP009762 AGAP002825 AGAP010592

**Software and algorithms**		

GraphPad Prism v.10	GraphPad Software	https://www.graphpad.com/
FlowJo v.10.10	FlowJo Software	https://www.flowjo.com/solutions/flowjo

**Other**		

Standard macrophage depletion kit (Clodrosome + Encapsome)	Encapsula NanoSciences	CLD-8901
Schneider’s insect medium	Sigma-Aldrich	S0146
Red fluorescent FluoSpheres (1.0 μm)	Thermo Fisher Scientific	F8821
DRAQ5	BD Biosciences	564903
ProLong Diamond antifade mountant with DAPI	Thermo Fisher Scientific	P36962
TURBO DNA-free kit	Thermo Fisher Scientific	AM2238
RevertAid first strand cDNA synthesis kit	Thermo Fisher Scientific	K1622
PowerUp SYBR Green master mix	Thermo Fisher Scientific	A25742
Aspirator	Sigma-Aldrich	A5177
Capillary tubes (3.5 Drummond # 3-000-203-G/X)	Fisher Scientific	21-171-4
Micropipette puller	Sutter Instrument	P-97
Neubauer hemocytometer slide	INCYTO	DHC-N01
Multitest slide	MP Biomedicals	096041805
Nanoject III injector	Drummond Scientific	3-000-207
Disposable pestle	Fisher Scientific	12-141-364
Motorized pestle mixer	Fisher Scientific	NC0493674
Bench-top centrifuge	Eppendorf	5417C
Bench-top centrifuge	Eppendorf	5804R
Digital dry baths/block heaters	Thermo Fisher Scientific	88870001
Digital chilling/heating dry baths	Torrey Pines Scientific	IC20
Water bath	Fisher Scientific	ISOTEM 210
pH meter	VWR	77619-150
Stainless steel needle (23 gauge)	Sigma-Aldrich	26272
Petri dish	Fisher Scientific	FB0875717
Microscope slides	Fisher Scientific	22-037-246
NanoDrop	Thermo Fisher Scientific	ND-2000
1.5 ml Eppendorf tubes	VWR	10025-726
0.2 ml PCR tube	VWR	470346-260
Thermocycler	MJ Research	PTC-100
QuantStudio 3 real-time PCR system	Thermo Fisher Scientific	A28140
Microscope	Nikon	Eclipse 50i
Fluorescent microscope	Zeiss Axio Imager	
Flow cytometry tube	Falcon	352057
Flow cytometer	BD Biosciences	BD FACSCanto

## Materials and equipment

**Table:** Anticoagulant buffer

Reagent	Final concentration (% v/v)	Amount
Schneider’s insect medium	60%	30 ml
Fetal bovine serum	10%	5 ml
Citrate buffer (98 mM NaOH, 186 mM NaCl, 1.7 mM EDTA, and 41 mM citric acid, pH 4.5)	30%	15 ml
**Total**		**50 ml**

## Step-by-step method details

### Injection of control and clodronate liposomes in adult female mosquitoes

**Timing: ∼3–4 h**

In this step, adult female mosquitoes are injected with clodronate liposomes to promote phagocyte depletion. The injection of control (empty) liposomes will be used for comparison.

1. Prepare 1:5 dilutions of control liposomes (LP) and clodronate liposomes (CLD) from the manufacturer stock solution using 1X PBS. A total volume of 25 μL (5 μL of liposomes, 20 μL of 1X PBS) should be adequate for most experiments. Dilutions can be mixed by pipetting, tapping, or inversion. Keep at room temperature, then mix again prior to use.

***Note:*** While previously published studies have used a 1:5 dilution to promote phagocyte depletion with minimal fitness effects,^1^^,^^9^^,^^10^ more recent studies demonstrate that dilutions of up to 1:50 can yield comparable results in phagocyte depletion.^13^

2. Prepare the injection setup.
  a. Turn on the chilling plate (Chilling/Heating Dry Bath) used for injections, setting the temperature to 4°C.
    ***Alternatives:*** Injections can also be performed using a setup in which mosquitoes are anaesthetized by CO_2_.
  b. Prepare a needle by filling with mineral oil and insert it into to the nanoinjector.
  c. Fill the needle with either the diluted LP or CLD using the nanoinjector.
  d. Set the injection volume on the nanoinjector to 69 nL.
3. Prepare the mosquitoes for injection.
  a. Place colony mosquitoes in a refrigerator at 4°C to cold anaesthetize.
  b. Transfer the cold anaesthetized mosquitoes to a petri dish placed on ice.
    ***Note:*** For larger experiments requiring increased mosquito numbers, smaller batches of mosquitoes should be used to limit the total time that they are kept at 4°C (on ice and on the chilling plate). Prolonged exposure to 4°C may reduce mosquito survival, especially when performing experiments with *Anopheles* species.
  c. Using forceps, gently transfer and position individual mosquitoes, ventral-side up, onto a glass slide placed on the chilling plate.
    ***Note:*** To enhance the speed of injections and minimize the time that the mosquitoes are cold anaesthetized, place several mosquitoes on the slide prior to injection (∼20–30 mosquitoes, depending on user preference). An experienced individual should take less than 5 min to inject ∼30 mosquitoes.
4. Inject each individual mosquito with 69 nL of LP or CLD by targeting the thoracic anepisternal cleft (middle of the lateral thorax) with the nanoinjector.

***Alternatives:*** If a Nanoject is unavailable, an alternative method using an aspirator equipped with a needle can be used for mosquito injections. While this technique is less accurate and more variable in the injection volume delivered per mosquito, it can be well-suited for initial or pilot studies to address phagocytic hemocyte function.

5. Transfer injected mosquitoes to a small enclosure (paper cup, cage) and allow for mosquitoes to recover under insectary conditions.

***Note:*** Previous studies have shown the CLD is able to promote phagocyte depletion by 12 h post-injection in *An. gambiae*,^13^ with phenotypes typically assessed at 24 h post-injection.^1^^,^^9^^,^^10^

### Confirmation of phagocytic hemocyte depletion using a hemocytometer

**Timing: ∼4 h**

In this step, use of a hemocytometer and hemocyte morphology will confirm phagocyte depletion following the injection of clodronate liposomes.

6. Prepare for hemolymph perfusions.
  a. Turn on the chilling plate used for perfusions, setting the temperature to 4°C.
  b. Use freshly prepared or thaw a frozen aliquot of anti-coagulant (AC) buffer.
  c. Cold anesthetize mosquitoes previously injected with LP or CLD by placing in a refrigerator at 4°C and transfer to a petri dish placed on ice.
7. Perform hemolymph perfusions on individual mosquitoes.
  a. Place an individual mosquito, positioned ventral-side up, on a glass slide placed on the chilling plate.
  b. Prior to perfusion, make an incision laterally on the mosquito between the seventh and eighth abdominal segments using a sterile 23-gauge hypodermic needle.
    **CRITICAL:** Avoid making an incision from the middle of abdomen where internal tissues such as the midgut, hindgut, and Malpighian tubules are located, this may result in microbial contamination of hemolymph samples or the isolation of non-target cells.
  c. Find the lateral side of the mesothorax and insert a glass needle filled with ∼10 μL of AC buffer. Slowly inject the AC buffer, delivering in intervals of ∼2 μL.
    ***Note:*** Perfusions can be performed using a variety of methods that include a Nanoject III, an aspirator, a 10 μL Hamilton syringe, or other type of injection setup.
    **CRITICAL:** The tip of the needle is extremely important. A smaller needle tip is desired to create adequate pressure for hemolymph perfusion without causing leakage at the injection site, yet the tip must be large enough to achieve a proper flow of the AC buffer. Buffer should not leak from the needle without applied pressure.
  d. Collect the perfused hemolymph from the incision in the abdomen immediately following the injection.
    ***Note:*** Hemolymph can be directly perfused onto the disposable Neubauer hemocytometer or indirectly transferred to the hemocytometer slide after collection with a P20 pipet using Sigmacote-treated pipet tips.
    ***Alternatives:*** A standard glass Neubauer hemocytometer can also be used, but needs to be carefully cleaned between each use.
8. Determine the proportion of hemocyte subtypes (prohemocyte, granulocyte and oenocytoid) based on morphology using a compound microscope.

***Note:*** To enhance rigor and reproducibility, count at least 200 hemocytes by light microscopy under a 40X objective. The use of phase contrast to enhance morphological phenotypes is recommended.

**CRITICAL:** Relatively low numbers of cells should be visible on the Neubauer slide. For example, when counting hemocytes in the four large squares (each containing 16 smaller squares), the total number of hemocytes typically varies between 200-300 cells. However, if the number of cells exceeds this number, this is likely due to cell debris or microbial contamination resulting from incision of the abdomen. Often numbers will exceed >1,000 cells with many small cell types (most likely bacteria) moving in the suspension. These samples should be discarded and the incision techniques further refined to minimize contamination.

9. Repeat for ∼8–10 mosquitoes per treatment. At least two independent biological experiments are recommended to produce reliable and statistically significant results.

***Note:*** Previous studies have suggested that the physiological status (i.e.- naive, blood-fed) of mosquitoes can influence the efficacy of phagocyte depletion.^1^ Following the above methodology, we have observed an ∼50% decrease in the percentage of granulocytes in naive mosquitoes, while we observe an ∼90% decrease in the percentage of granulocytes following blood-feeding.

### Confirmation of phagocytic hemocyte depletion using immunofluorescence

**Timing: ∼24 h**

In this step, immunofluorescence experiments will confirm phagocyte depletion following the injection of clodronate liposomes.

10. *In vivo* labeling of hemocytes with CM-DiI.
  a. Turn on the chilling plate used for perfusions, setting the temperature to 4°C.
  b. Cold anesthetize mosquitoes previously injected with LP or CLD by placing in a refrigerator at 4°C and transfer to a petri dish placed on ice.
  c. Using forceps, gently transfer and position individual mosquitoes, ventral-side up, onto a glass slide placed on the chilling plate.
  d. As previously outlined (Steps 2–4), inject mosquitoes using the Nanoject III to deliver 138 nL of 100 μM CM-DiI intrathoracically to each individual mosquito.
    ***Note:*** CM-DiI is a lipophilic dye that specifically labels mosquito hemocytes.^14^^,^^15^ The labeling of hemocytes is the most efficient *in vivo* following injection.
  e. Transfer mosquitoes to an enclosed container and allow the mosquitoes to recover by incubating at 27°C for 20 min.
11. Perfuse hemolymph perfusions as outlined above (Step 7). However, instead of collecting hemolymph for analysis with a hemocytometer, perfuse hemolymph directly onto a multitest glass slide.

***Note:*** An advantage of the multitest slides is the ability to perform immunofluorescence assays (IFAs) for multiple individual mosquitoes on a single slide.

12. Perform IFA experiments on hemocytes from LP- and CLD-treated mosquitoes.
  a. Place the glass slide in a pipette tip box and allow hemocytes to adhere to the slide for 30 min at room temperature (RT).
    **CRITICAL:** Avoid letting the perfused hemolymph dry out on the slide. It is suggested that slides should be placed inside a container (such as an empty pipette tip box) to avoid drying out. In addition, a damp paper towel can be placed at the bottom of the container to increase the humidity to further prevent the hemolymph from evaporating.
  b. After incubation, fix hemocytes by adding 50 μL of 4% paraformaldehyde (PFA) to each well for 30 min at RT.
    ***Note:*** Add the PFA directly to the remaining hemolymph. This maximizes the adherence of cells to the slide. Removing the remaining hemolymph or washing prior to fixation has the potential to reduce cell retention on the slide.
  c. Remove the PFA and wash three times for 5 min with 1X PBS.
  d. Block each well by treating with 50 μL of 1% BSA in 1X PBS for 30 min.
  e. Treat each well with 50 μL of FITC-conjugated WGA (1:500 in 1% BSA, 1X PBS) and incubate overnight (∼16 h) at 4°C.
    ***Note:*** WGA is a lectin that recognizes N-acetylglucosamine modifications on membrane-bound glycoproteins. Previous studies demonstrate that WGA universally stains mosquito hemocytes.^1^^,^^16^^,^^17^
  f. Remove the WGA stain and wash the slides three times for 5 min in 1X PBS.
  g. Add antifade mounting media containing DAPI (such as ProLong Diamond Antifade Mountant with DAPI) to label nuclei and to protect against photobleaching when performing microscopy.
    ***Alternatives:*** Nuclei can be labeled through a variety of methods. One alternative is to use Hoechst 33342, which can be mixed in with the CM-DiI injections outlined above (Step 10) to efficiently label hemocyte nuclei.^14^^,^^15^
  h. Add a cover slip and adhere using nail polish.
13. Examine IFAs of hemocyte populations collected from LP- and CLD-treated mosquitoes using a compound fluorescence microscope.
  a. Hemocytes can be visualized by red (CM-DiI), green (FITC-WGA), and blue (DAPI, Hoechst) fluorescence.
  b. Sample treatments can be compared by counting the total number of hemocytes attached to the slides for each individual mosquito or as representative images to display visible differences in cell number.

***Note:*** CM-DiI does not label all hemocytes, hence the use of the FITC-WGA to act as an additional counterstain. However, CM-DiI^+^ cells are highly susceptible to clodronate liposome treatment and should be reduced in their abundance.

### Confirmation of phagocytic hemocyte depletion by gene expression analysis

**Timing: 2 days**

In this step, use of gene expression analysis via real-time qPCR will confirm phagocyte depletion following the injection of clodronate liposomes.

14. Isolate total RNA from LP- or CLD-treated mosquitoes.
  a. One day post-injection of LP or CLD, collect ∼10 individual mosquitoes in a 1.5 ml Eppendorf tube for RNA isolation. The samples can be either stored at −80°C or immediately processed.
  b. Add 500 μL of TRIzol reagent to each sample and homogenize with a motorized pestle until any large tissues are gone.
  c. Add an additional 500 μL of TRIzol reagent to the homogenized samples and incubate for 5 min at RT.
  d. Centrifuge samples at 12,000 x g for 15 min at 4°C using a refrigerated centrifuge and transfer the supernatant to a new 1.5 ml tube.
  e. Add 200 μL of chloroform to each sample and shake vigorously for 15 s.
  f. Incubate samples at RT for 2 to 3 min, then centrifuge at 12,000 x g for 15 min at 4°C.
  g. Remove the upper aqueous clear layer and transfer to a new 1.5 ml tube.
  h. Add 250 μL of isopropyl alcohol and 250 μL of 0.8 M sodium citrate/1.2 M NaCl. Incubate samples at RT for 10 min.
    **CRITICAL:** The addition of the 0.8 M sodium citrate/ 1.2 M NaCl solution is absolutely necessary for whole mosquito samples. Without this step pigments from the whole mosquito lysate will form a reddish-brown RNA pellet that will interfere with RNA quantification and downstream PCR steps.
  i. Centrifuge samples at 12,000 x g for 10 min at 4°C. When complete, a small whitish RNA pellet should be visible. Carefully remove all of the supernatant using a narrow bore pipette tip.
  j. Wash the RNA pellet using 500 μL of 70% of ethanol and centrifuge at 7,500 x g for 5 min at 4°C. Repeat this step.
  k. Remove the ethanol supernatant and air dry the RNA pellet for 5–10 min.
  l. Resuspend RNA pellets with 40 μL of RNase-free water. Vortex samples to completely dissolve pellets.
    ***Note:*** Samples can be optionally incubated at 65°C using a water bath or heating block to help resuspend the RNA pellets.
  m. Treat the RNA sample with DNase to remove any gDNA present by adding 0.1 volume 10X TURBO DNase buffer and 1 μL of TURBO DNase. Incubate in a 37°C water bath for 30 min.
  n. Add DNase inactivation reagent (0.1 volume) and incubate at RT for 5 min.
  o. Centrifuge at 12,000 x g for 2 min at 4°C. Transfer the RNA (supernatant) to a clean 1.5 ml tube.
  p. Measure the RNA concentration using a NanoDrop spectrophotometer.
    Store the RNA samples at −80°C or proceed directly to cDNA synthesis.
15. Perform cDNA synthesis using the RevertAid First Strand cDNA Synthesis kit according to the manufacturer’s protocol.
  a. Add 2 μg of total RNA (volume will vary), 1 μL of 100 μM oligo dT, 1 μL of 100 μM random hexamer and nuclease free water to a total of 12 μL in a 0.2 ml PCR tube.
  b. Incubate the mixture at 65°C for 5 min using a thermocycler. Quickly spin in a PCR mini centrifuge and chill on ice.
  c. Add 4 μL of 5X reaction buffer, 1 μL of RNase inhibitor, 2 μL of 10 mM dNTPs and 1 μL of reverse transcriptase to each sample. Incubate at RT (∼25°C) for 5 min.
  d. Incubate samples at 42°C for 1 h, then 70°C for 5 min to terminate the reaction.
  e. Dilute the cDNA (1:5) in nuclease free water and keep at −20°C.
16. Perform real-time qPCR analysis from the prepared cDNA of LP- and CLD-treated mosquitoes.
  a. Evaluate the expression of *Eater* and *Nimrod B2*, genes expressed in phagocytic granulocyte populations in *An. gambiae*,^1^^,^^9^ to serve as an indirect method to evaluate cell numbers.
  b. The inclusion of *rpS7* (or similar housekeeping gene) should be included as an internal reference gene to standardize gene expression between samples.
    ***Note:*** The use of an additional gene, such as *PPO1*, can also be included to represent non-target oenocytoid populations.^10^
  c. Prepare the real-time qPCR reaction with PowerUp SYBRGreen Master Mix using a QuantStudio 3 Real-Time PCR System.

***Note:*** Prepare a larger volume of real-time qPCR master mix than needed for all of the reactions to prevent running out of the mixture during pipetting. This will produce more consistent and accurate results between sample replicates.

**Table:** Real-time qPCR cycling condition

Steps	Temperature	Time	Cycles
Initial Denaturation	95 °C	10 min	1
Denaturation	95 °C	15 s	40 cycles
Annealing	65 °C	1 min	
Hold	4 °C	Forever	

### Confirmation of phagocytic hemocyte depletion using flow cytometry analysis

**Timing: ∼2 days**

In this step, use of flow cytometry will confirm phagocyte depletion following the injection of clodronate liposomes.

17. Examine LP- and CLD-treated mosquitoes by flow cytometry.
  a. One day post-injection of LP or CLD, cold anesthetize mosquitoes and place them in a petri dish plate on ice.
  b. Inject mosquitoes with 69 nL of red fluorescent carboxylate-modified microspheres (1 μm) using a final concentration of 2% (vol/vol) in 1X PBS. This enables the detection of phagocytic granulocyte populations by flow cytometry based on their ability to phagocytose the fluorescent beads.^1^^,^^18^ Allow for mosquitoes to recover for 30 min at 27°C.
  c. As outlined in Step 7, perfuse hemolymph from ∼40 individual mosquitoes from each treatment with AC buffer. Collect hemolymph directly in 1.5 ml microcentrifuge tubes kept on ice.
  d. Fix cells by the addition of a 1:1 volume of 4% paraformaldehyde. Incubate for 1 h at 4°C, then centrifuge samples at 2000 x g at 4°C for 5 min. Discard the supernatant.
  e. Wash the samples in 1 ml of 1X PBS, then centrifuge samples at 2000 x g at 4°C for 5 min. Repeat the wash step.
    ***Note:*** Carefully perform these wash steps. Remove ∼90% of the supernatant to avoid potentially disposing of the samples. A weak cell pellet may not always be visible.
  f. Stain cell samples with FITC-WGA (1:5000) and DRAQ5 (1:1000) in 1X PBS. Incubate overnight (∼16 h) at 4°C.
  g. After incubation, centrifuge cells and discard the supernatant.
  h. Wash two times in 1X PBS and centrifuge as described above to remove excess stain.
  i. Add 200 μL of 1X PBS and transfer to flow cytometry tubes.
  j. Perform flow cytometry using a BD FACSCanto cytometer or comparable instrument.

## Expected outcomes

Herein, we outline multiple techniques that can be employed for the use and validation of clodronate-mediated depletion of phagocytic hemocytes in *An. gambiae*, as well as other arthropod systems with slight modification. We outline multiple methods relying on microscopy, real-time qPCR, or flow cytometry that offer flexibility for users to choose appropriate means of validation that can vary by experimental design and the availability of technical resources. For the purpose of rigor and reproductivity, we believe that more than one of these techniques should be used to evaluate phagocyte depletion, although individual users can determine how to apply each of these methodologies for validation.

**Figure 1:**
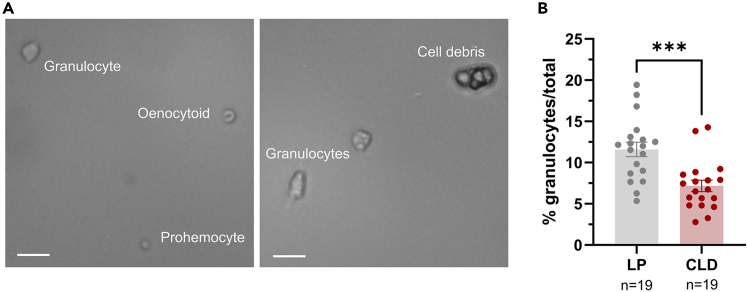
Validation of granulocyte depletion using a hemocytometer Examples of hemocyte subtypes when visualized on a hemocytometer using light microscopy (A). Granulocytes display distinct morphological characteristics based on their adherent properties and larger size. Both prohemocytes and oenocytoids are round, with oenocytoids distinguished by their relatively larger size and cupped-like appearance. Cell debris, such as fat body cells, can occasionally be observed and distinguished as clusters of large cells that are distinct from the morphology of the mosquito hemocyte subtypes. The percentage of granulocytes from control liposome (LP) or clodronate liposome (CLD) treatments can be assessed to confirm the depletion of granulocytes at 24 h post-injection (B). Error bars represent the mean ± SEM of three independent replicates. Data were tested for normality and analyzed using an unpaired t-test *(∗∗∗P < 0.001*) using GraphPad Prism 10. Scale bar, 10 μm.

In perfused hemolymph samples observed under a hemocytometer, phagocytic hemocytes (granulocytes) exhibit distinct morphological characteristics, exhibiting a larger size and displaying adherence to the slide with a more spread form (often with pseudopodia) compared to other hemocyte types (such as prohemocyte and oenocytoid). Consequently, a significant reduction can be observed in the proportion of granulocytes in clodronate liposome (CLD) mosquitoes compared to those treated with control liposomes (LP). Phenotypes should be evaluated from 15–20 individual mosquitoes (Figure 1).^1^^,^^2^^,^^13^

**Figure 2:**
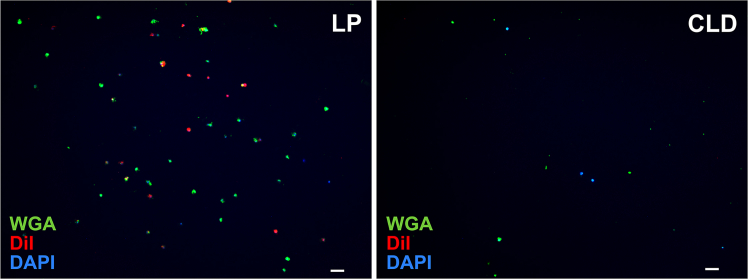
Confirmation of phagocyte depletion by IFA after clodronate liposome treatment Immunofluorescence assays (IFAs) were performed to examine perfused hemocytes from control liposome (LP) or clodronate liposome (CLD) treatments. Attached hemocytes are visualized using FITC-WGA (green), CM-DiI (red) and DAPI (blue), to clearly demonstrate the reduced number of attached granulocytes in CLD-treated mosquitoes. Scale bar, 20 μm.

The depletion of phagocytic granulocyte populations can also be validated using immunofluorescence assays (IFAs) using known hemocyte stains such as CM-DiI and FITC-labeled wheat germ agglutinin (WGA) that are paired with DAPI or other DNA stains to visualize cell nuclei. Hemocyte numbers from LP- and CLD-treated mosquitoes can be directly quantified to determine whether granulocyte numbers are significantly reduced or visualized by micrographs that visually display differences in cell number (Figure 2).

**Figure 3:**
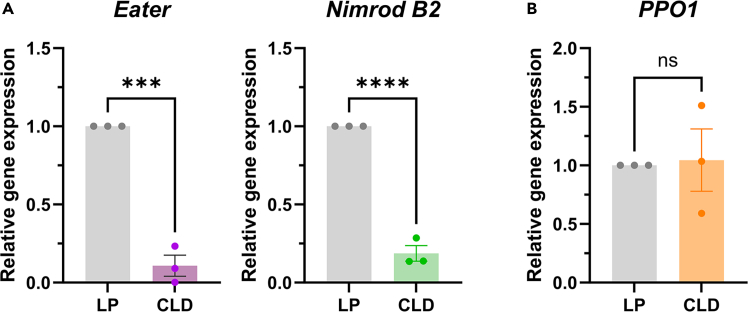
Confirmation of phagocytic hemocyte depletion using real-time qPCR analysis The depletion of phagocytic hemocytes was confirmed by assessing the expression of the phagocytic cell markers, *Eater* and *Nimrod B2*, in control liposome (LP)- or with clodronate liposome (CLD)-treated mosquitoes (A). In contrast, *PPO1*, a marker for non-phagocytic hemocytes, displayed no difference in expression between LP- and CLD-treated groups (B). Bars represent the mean ± SEM of three independent biological replicates (dots). Statistical analysis was performed using an unpaired *t*-test in GraphPad Prism 10.0. Asterisks indicate statistical significance (∗∗∗, *P* < 0.001; ∗∗∗∗, *P* < 0.0001). ns, not significant.

Molecular validation of phagocyte depletion as a result of CLD treatment can be assessed using real-time qPCR by examining the expression of with specific hemocyte markers such as *Eater* and *Nimrod B2*.^1^ These genes are enriched in phagocytic granulocytes,^9^ such that their expression can by proxy examine granulocyte numbers.^1^ In addition, non-phagocytic markers like *PPO1* can be used to confirm that the effects of CLD are specific to phagocytic hemocytes and would unchanged across sample treatments (Figure 3).

**Figure 4:**
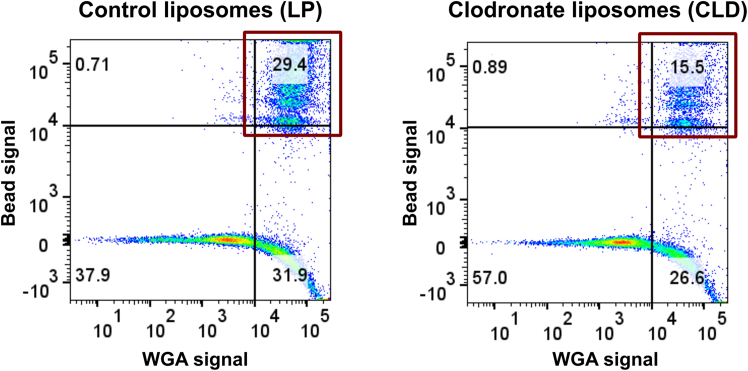
Assessment of phagocytic hemocyte proportions using flow cytometry The proportion of phagocytic hemocytes was evaluated between control liposome (LP) and clodronate liposome (CLD) treatments using flow cytometry. Double-positive hemocytes were identified by gating based on fluorescent signal thresholds: red for bead uptake and green for WGA staining, located in the upper right quadrant (denoted by red box). The impact of CLD on phagocytic hemocyte depletion was assessed by comparing the percentage of double-positive (phagocytic) hemocytes between the LP and CLD samples (29.4 vs. 15.5). This example displays an approximate 47% reduction (29.4–15.5 / 29.4) in the number of phagocytic cells following CLD treatment under naïve conditions.

Further validation can be performed using flow cytometry analysis to impartially examine phagocytic hemocyte depletion. Aided by the use of FITC-labeled WGA and DRAQ5 to stain hemocytes or nuclei respectively, phagocytic granulocyte depletion can be examined by comparing the proportion of phagocytic cells that have taken up red fluorescent beads and assessed using FlowJo software^1^ (Figure 4). While the percentage of cells may vary between techniques, the reduction in phagocytic hemocytes observed through flow cytometry is consistent with results obtained from manual cell counting using a hemocytometer.

## Quantification and statistical analysis

### Hemocyte counts using a hemocytometer or IFA

Data from individual mosquitoes should be pooled per treatment. Data should be tested for normality, with subsequent statistical analysis performed on individual comparisons using either a Student’s t-test (parametric) or a Mann-Whitney (non-parametric) test using GraphPad Prism. Multiple comparisons should utilize a one-way ANOVA (parametric) or Kruskal-Wallis (non-parametric) test.

### Gene expression analysis

Real-time qPCR results should be normalized to rpS7 as an internal reference, with target gene expression examined using the 2^−ΔΔCt^ method.^19^ Independent biological replicates should be combined and examined using a Student’s t-test to determine significance using GraphPad Prism.

**Assessment of phagocytic hemocytes by gating WGA and bead positive cells using the FlowJo software** (Detailed gating descriptions can be found in Figure S6 of Kwon and Smith, 2019^1^)

1. File → Open.
2. Select→fcs files (unstained hemocytes, a red fluorescent bead only, LP and CLD samples).
3. Adjust events by using threshold values for gating as determine the red bead size by forward scatter channel (FSC).
4. Use unstained cells to determine cutoffs for positive WGA and DRAQ5 signals to remove any autofluorescence background.
5. Select double positive (bead and WGA)
6. Determine the percentage of phagocytic hemocytes in LP and CLD samples to measure the effect of CLD depletion.

## Limitations

Differentiating hemocyte subtypes based on morphology can be challenging and often contentious due to the lack of defined hemocyte markers.^20^ Therefore, the outlined microscopy-based hemocytometer experiments will have some user-based variability in how they are quantified. In addition, previous studies suggest that CLD may not act on all phagocytic hemocyte populations.^1^ With evidence supporting the presence of multiple phagocytic granulocyte populations,^1^^,^^9^^,^^12^^,^^21^ it is currently unclear which granulocyte subpopulations may be specifically targeted by CLD.

## Troubleshooting

### Problem 1

Poor mosquito survival after injection (Relevant to step 3).

### Potential solution

• Mosquito intrathoracic injections require significant practice to ensure that a high percentage of mosquitoes survive. The bore of the needle should be as small as possible, while still being able to deliver liquid, and should only minimally penetrate the mosquito cuticle during the injection. Prior to experiments, researchers should practice by injecting sterile 1x PBS or other non-toxic dyes/food coloring to achieve survival rate more than 90% before proceeding.
• Maintain a clean injection setup. Sterilize equipment with 70% ethanol prior to use. Condensation on the cold plate can potentially introduce microbes directly into injection site and should be prevented as best as possible.

### Problem 2

Poor recovery of hemocytes in the hemocytometer or IFA experiments (Relevant to steps 7, 8, and 13).

### Potential solution

• Check the preparation of the anticoagulant buffer, maintaining the correct pH is crucial for hemocyte stability.
• Practice the perfusions, aiming for reliability and consistency between individual mosquitoes. Make sure that the bore of the needle is as small as possible to create enough pressure to properly flush the hemolymph without leakage at the site of injection.

### Problem 3

There is an abundance of cell debris and microbial contamination when perfused hemolymph is examined using a hemocytometer (Relevant to steps 7 and 8).

### Potential solution

• Mosquito wings can be removed prior to the incision and perfusion process to prevent inadvertent contact between the wings and the perfusion droplet during expulsion.
• The incision should be as “clean” as possible. Additional damage or disruption at the incision site can introduce cell debris in the perfusion.
• The incision for the perfusion may have penetrated an internal tissue, such as the midgut or hindgut, that introduced significant microbial contamination in the perfusion.

### Problem 4

Effects of CLD treatment on phagocytic hemocyte depletion are minimal (Relevant to steps 8, 13, 16, and 17).

### Potential solution

• Hemocyte numbers quickly decline in adult mosquitoes and are believed to contribute to age-associated mortality.^22^ Therefore, using mosquitoes between 3- and 5-days post-emergence is recommended for experiments to minimize the potential age-related effects of phagocytic hemocyte depletion.

### Problem 5

Low number of cells/events in flow cytometry analysis (Relevant to step 17).

### Potential solution

• Increase the sample size of mosquitoes used in the flow cytometry analysis.
• Reduce the washing and incubation times during processing of the samples. While the FITC-WGA staining can be helpful in denoting hemocytes, it is not absolutely necessary and can be removed. This modification should reduce the total number of wash steps and incubation times, which may lead to hemocyte loss and compromise the accuracy of phagocytic hemocyte quantification.
• Ensure that the gating strategy is accurate and does not exclude potential populations of hemocytes.

## Resource availability

### Lead contact

Further information and requests for additional resources should be addressed to Ryan Smith (smithr@iastate.edu).

### Technical contact

Technical questions on executing this protocol should be addressed to either Hyeogsun Kwon (hskwon@iastate.edu) or Ryan Smith (smithr@iastate.edu).

### Materials availability

This study did not generate any new or unique resources. All described reagents are commercially available.

### Data and code availability

This study did not generate/analyze any large datasets or code. Raw source data presented in the figures in the paper are available from the corresponding authors on request.

## Acknowledgments

Support was provided by the National Institute of Allergy and Infectious Diseases of the National Institutes of Health under award number AI177540 to R.C.S.

## Author contributions

H.K. conceived the project, developed the methodology, designed the graphical abstract, and wrote the initial draft. R.C.S. conceived the project, contributed to the methodology, and edited the manuscript.

## Declaration of interests

The authors declare no competing interests.
